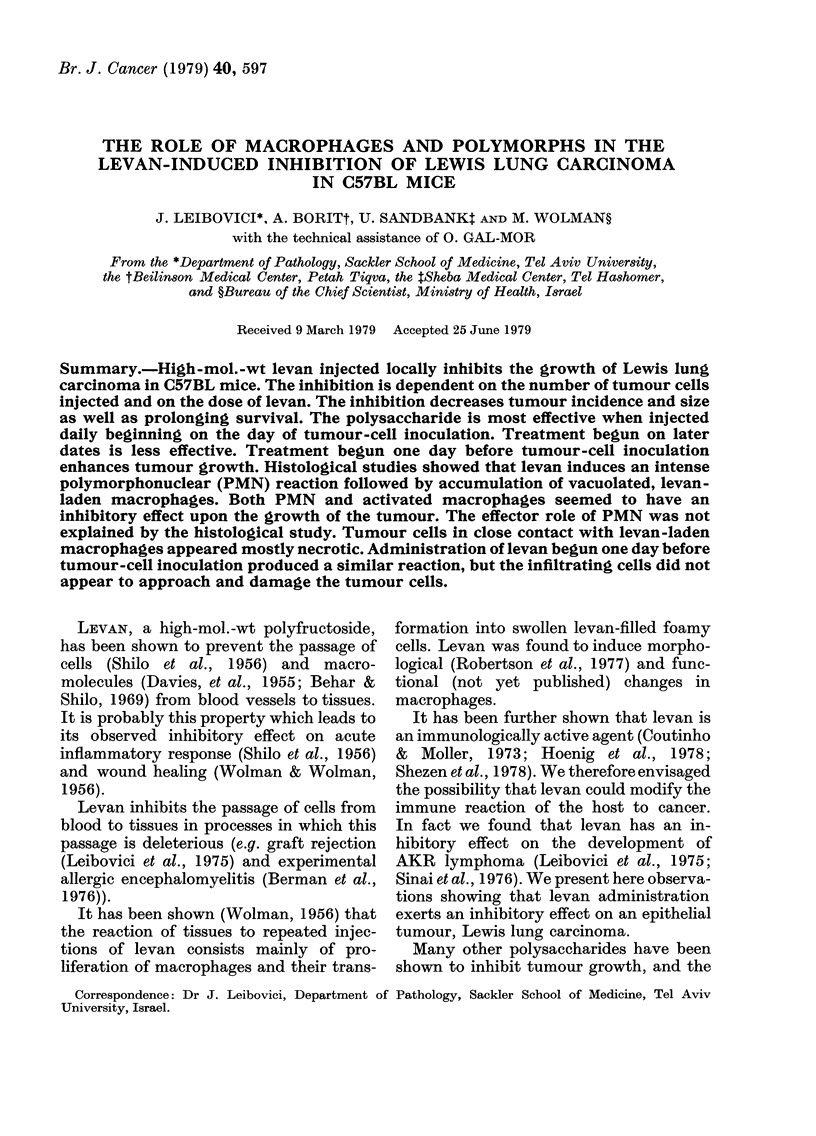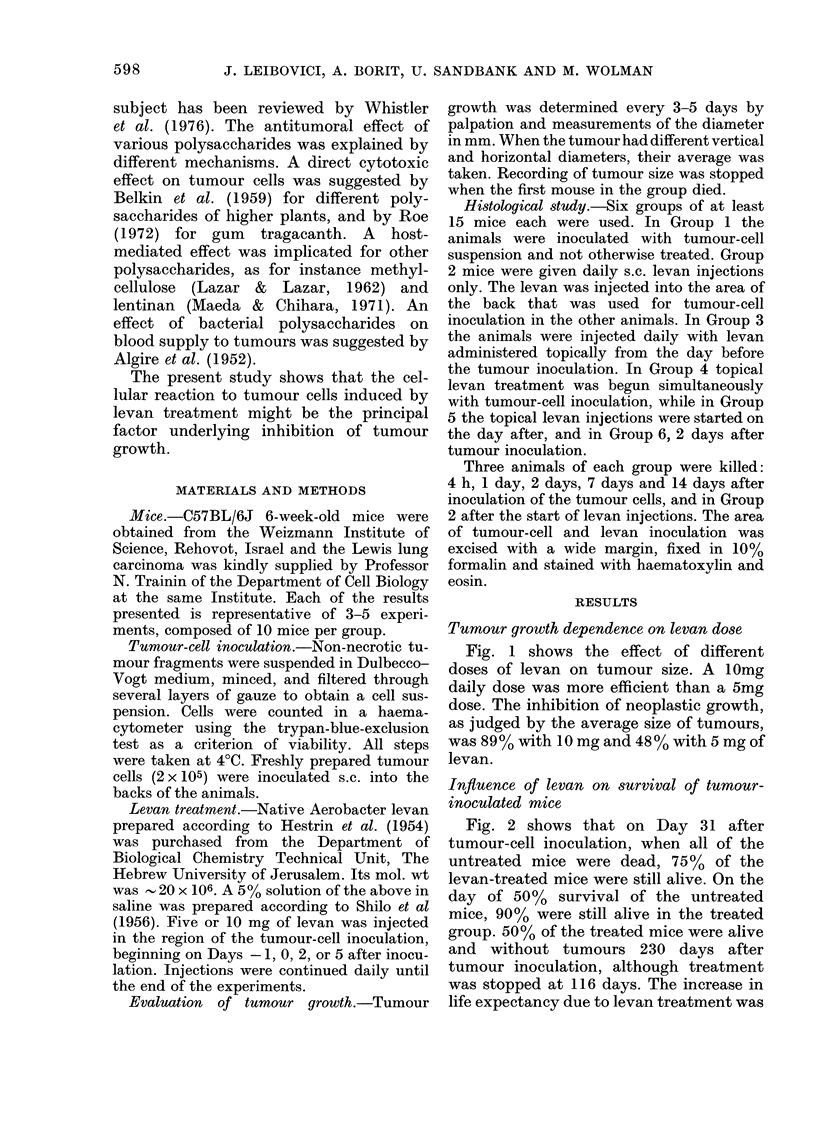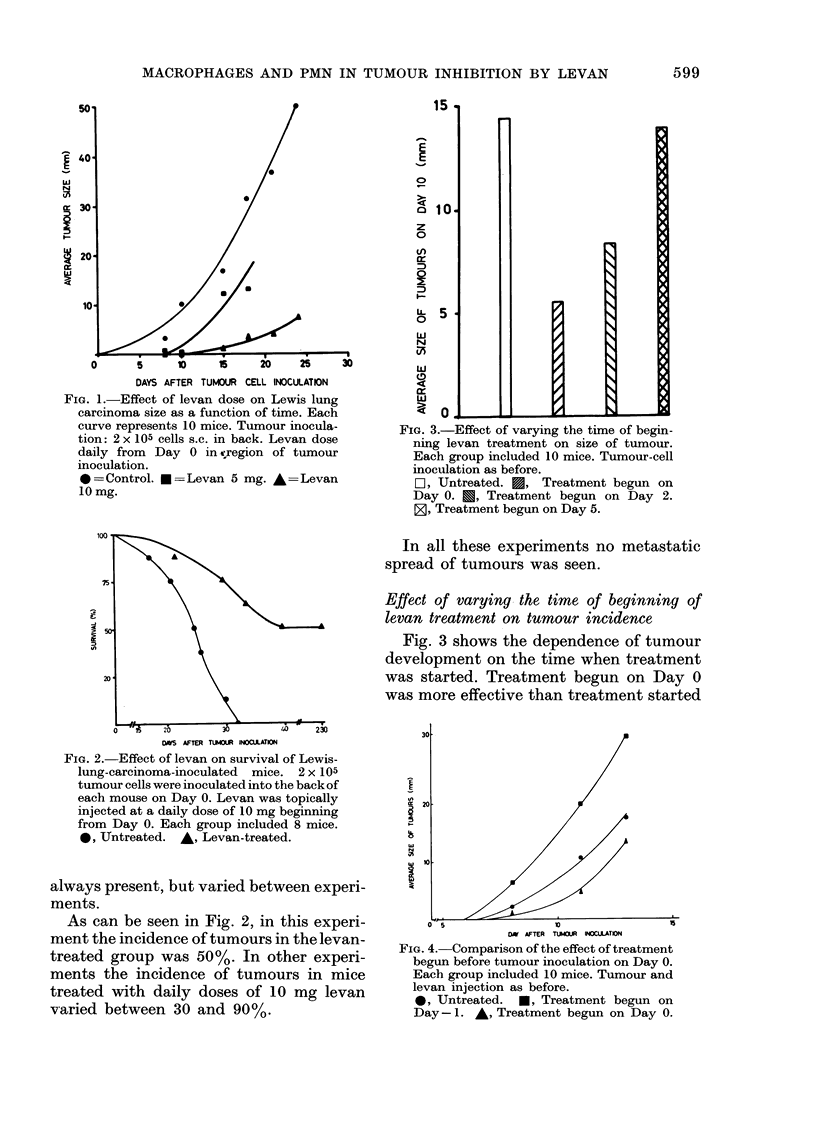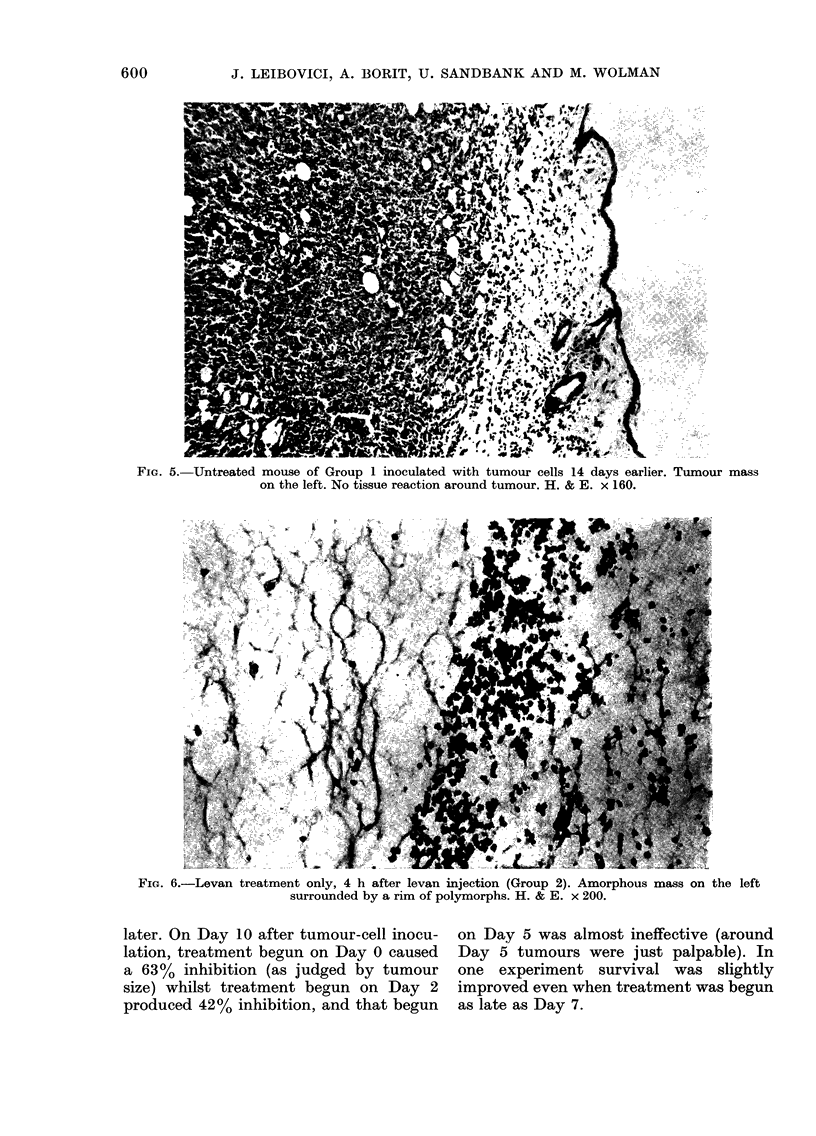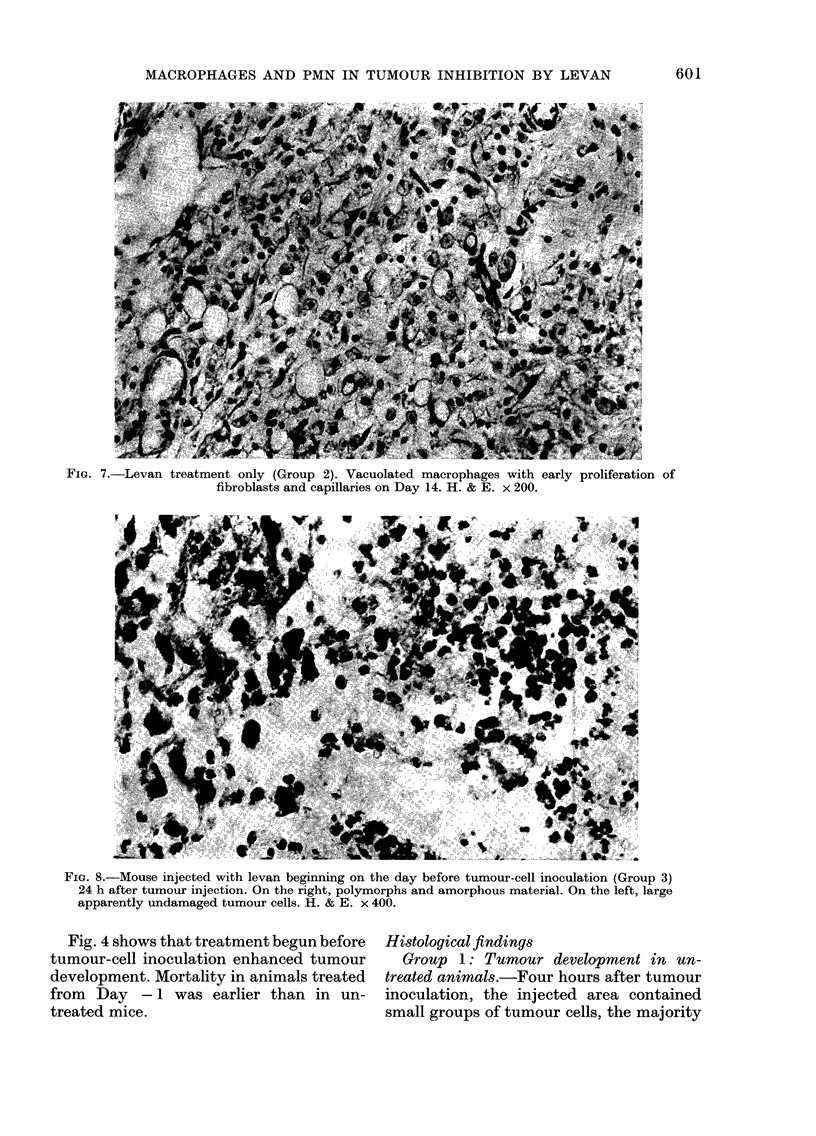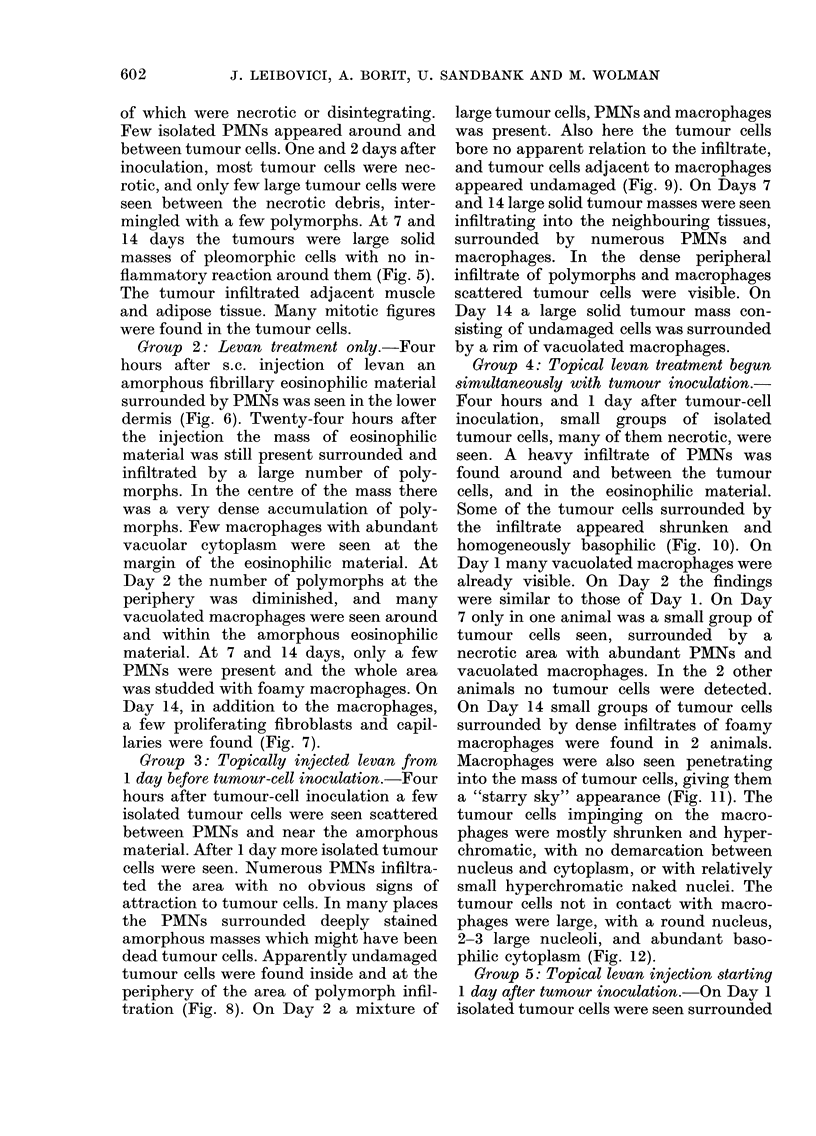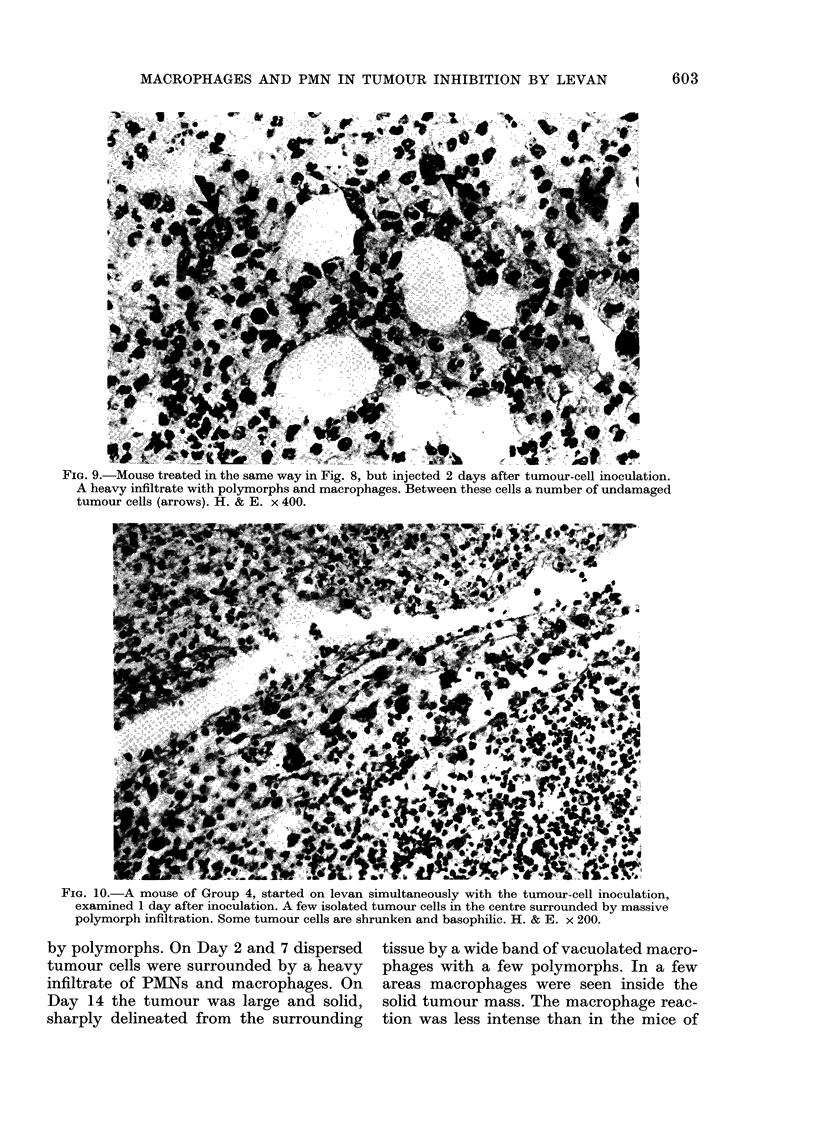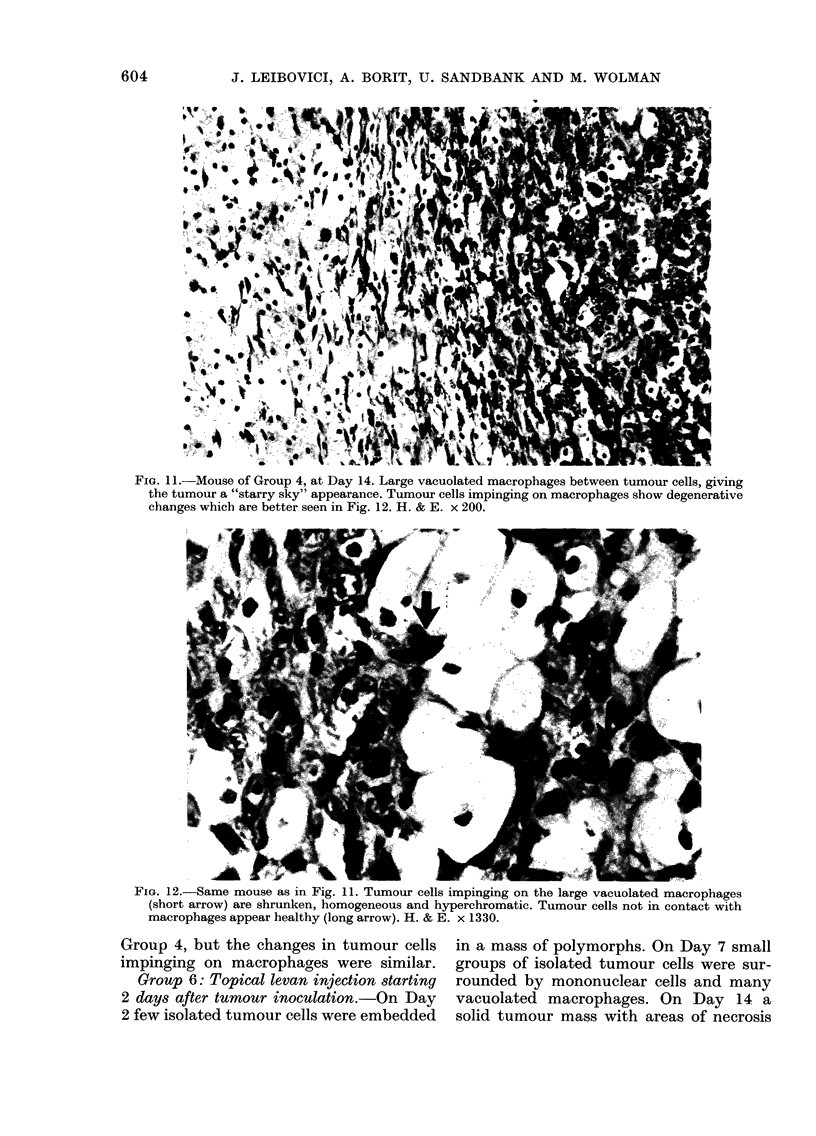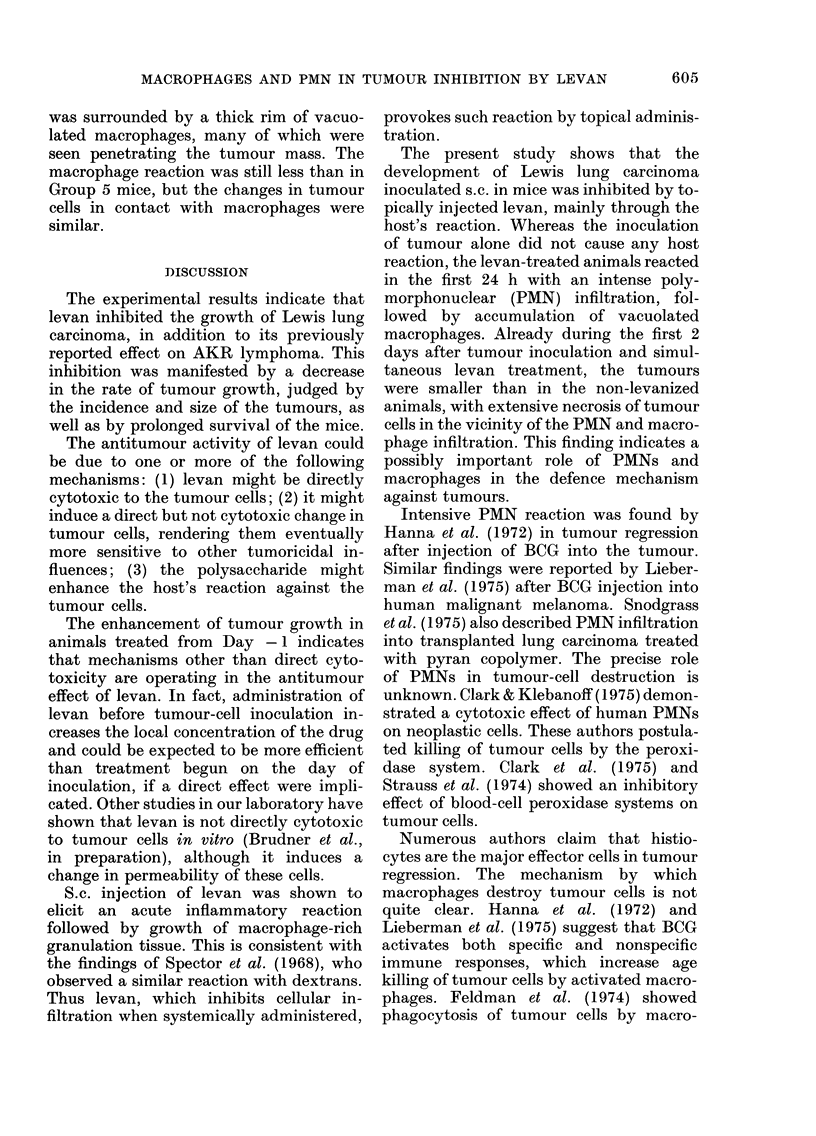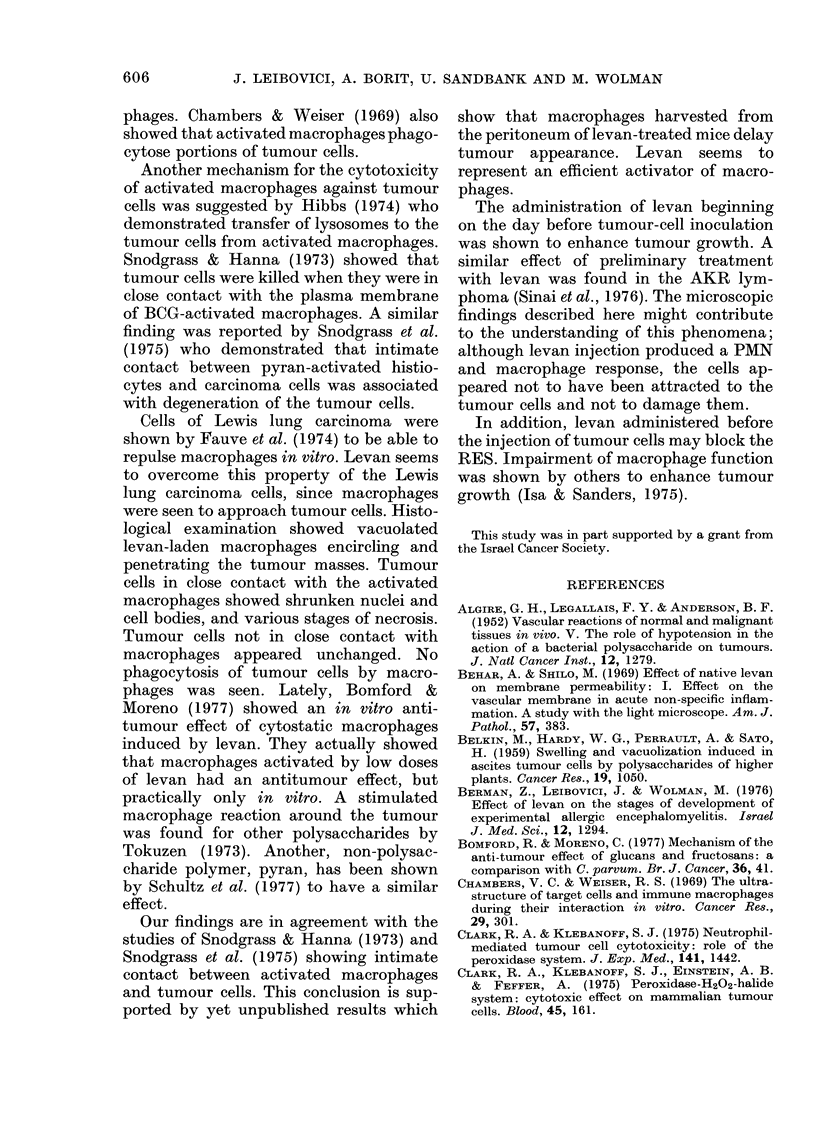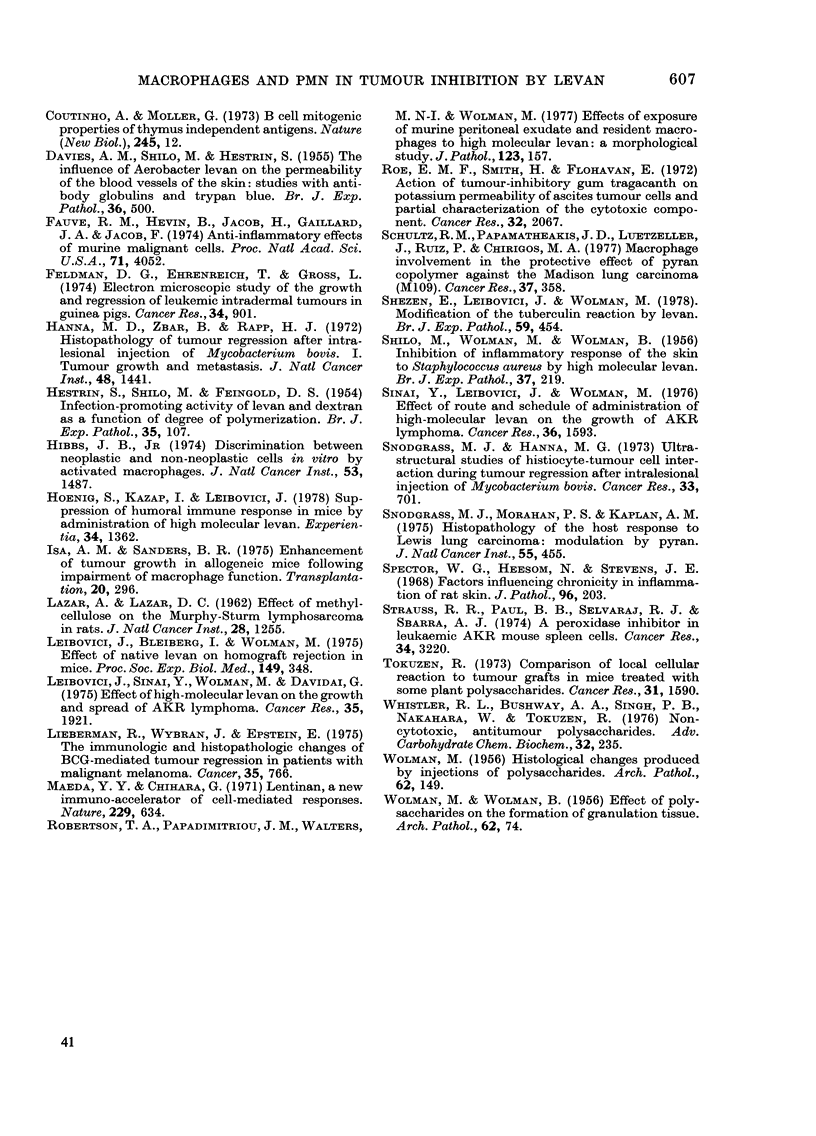# The role of macrophages and polymorphs in the levan-induced inhibition of Lewis lung carcinoma in C57BL mice.

**DOI:** 10.1038/bjc.1979.225

**Published:** 1979-10

**Authors:** J. Leibovici, A. Borit, U. Sandbank, M. Wolman

## Abstract

**Images:**


*Br. J. Cancer* (1979) **40**, 597

### THE ROLE OF MACROPHAGES AND POLYMORPHS IN THE LEVAN-INDUCED INHIBITION OF LEWIS LUNG CARCINOMA IN C57BL MICE

J. LEIBOVICI*, A. BORIT†, U. SANDBANK‡ AND M. WOLMAN§
with the technical assistance of O. GAL-MOR

*From the *Department of Pathology, Sackler School of Medicine, Tel Aviv University, the †Beilinson Medical Center, Petah Tiqva, the ‡Sheba Medical Center, Tel Hashomer, and §Bureau of the Chief Scientist, Ministry of Health, Israel*

Received 9 March 1979 Accepted 25 June 1979

**Summary.—High-mol.-wt levan injected locally inhibits the growth of Lewis lung carcinoma in C57BL mice. The inhibition is dependent on the number of tumour cells injected and on the dose of levan. The inhibition decreases tumour incidence and size as well as prolonging survival. The polysaccharide is most effective when injected daily beginning on the day of tumour-cell inoculation. Treatment begun on later dates is less effective. Treatment begun one day before tumour-cell inoculation enhances tumour growth. Histological studies showed that levan induces an intense polymorphonuclear (PMN) reaction followed by accumulation of vacuolated, levan-laden macrophages. Both PMN and activated macrophages seemed to have an inhibitory effect upon the growth of the tumour. The effector role of PMN was not explained by the histological study. Tumour cells in close contact with levan-laden macrophages appeared mostly necrotic. Administration of levan begun one day before tumour-cell inoculation produced a similar reaction, but the infiltrating cells did not appear to approach and damage the tumour cells.**

LEVAN, a high-mol.-wt polyfructoside, has been shown to prevent the passage of cells (Shilo *et al.*, 1956) and macromolecules (Davies, *et al.*, 1955; Behar & Shilo, 1969) from blood vessels to tissues. It is probably this property which leads to its observed inhibitory effect on acute inflammatory response (Shilo *et al.*, 1956) and wound healing (Wolman & Wolman, 1956).

Levan inhibits the passage of cells from blood to tissues in processes in which this passage is deleterious (*e.g.* graft rejection (Leibovici *et al.*, 1975) and experimental allergic encephalomyelitis (Berman *et al.*, 1976)).

It has been shown (Wolman, 1956) that the reaction of tissues to repeated injections of levan consists mainly of proliferation of macrophages and their transformation into swollen levan-filled foamy cells. Levan was found to induce morphological (Robertson *et al.*, 1977) and functional (not yet published) changes in macrophages.

It has been further shown that levan is an immunologically active agent (Coutinho & Moller, 1973; Hoenig *et al.*, 1978; Shezen *et al.*, 1978). We therefore envisaged the possibility that levan could modify the immune reaction of the host to cancer. In fact we found that levan has an inhibitory effect on the development of AKR lymphoma (Leibovici *et al.*, 1975; Sinai *et al.*, 1976). We present here observations showing that levan administration exerts an inhibitory effect on an epithelial tumour, Lewis lung carcinoma.

Many other polysaccharides have been shown to inhibit tumour growth, and the

Correspondence: Dr J. Leibovici, Department of Pathology, Sackler School of Medicine, Tel Aviv University, Israel.

subject has been reviewed by Whistler *et al.* (1976). The antitumoral effect of various polysaccharides was explained by different mechanisms. A direct cytotoxic effect on tumour cells was suggested by Belkin *et al.* (1959) for different polysaccharides of higher plants, and by Roe (1972) for gum tragacanth. A host-mediated effect was implicated for other polysaccharides, as for instance methylcellulose (Lazar & Lazar, 1962) and lentinan (Maeda & Chihara, 1971). An effect of bacterial polysaccharides on blood supply to tumours was suggested by Algire *et al.* (1952).

The present study shows that the cellular reaction to tumour cells induced by levan treatment might be the principal factor underlying inhibition of tumour growth.

### MATERIALS AND METHODS

*Mice.*—C57BL/6J 6-week-old mice were obtained from the Weizmann Institute of Science, Rehovot, Israel and the Lewis lung carcinoma was kindly supplied by Professor N. Trainin of the Department of Cell Biology at the same Institute. Each of the results presented is representative of 3–5 experiments, composed of 10 mice per group.

*Tumour-cell inoculation.*—Non-necrotic tumour fragments were suspended in Dulbecco–Vogt medium, minced, and filtered through several layers of gauze to obtain a cell suspension. Cells were counted in a haemacytometer using the trypan-blue-exclusion test as a criterion of viability. All steps were taken at 4°C. Freshly prepared tumour cells ($2 \times 10^5$) were inoculated s.c. into the backs of the animals.

*Levan treatment.*—Native Aerobacter levan prepared according to Hestrin *et al.* (1954) was purchased from the Department of Biological Chemistry Technical Unit, The Hebrew University of Jerusalem. Its mol. wt was $\sim 20 \times 10^6$. A 5% solution of the above in saline was prepared according to Shilo *et al* (1956). Five or 10 mg of levan was injected in the region of the tumour-cell inoculation, beginning on Days −1, 0, 2, or 5 after inoculation. Injections were continued daily until the end of the experiments.

*Evaluation of tumour growth.*—Tumour growth was determined every 3–5 days by palpation and measurements of the diameter in mm. When the tumour had different vertical and horizontal diameters, their average was taken. Recording of tumour size was stopped when the first mouse in the group died.

*Histological study.*—Six groups of at least 15 mice each were used. In Group 1 the animals were inoculated with tumour-cell suspension and not otherwise treated. Group 2 mice were given daily s.c. levan injections only. The levan was injected into the area of the back that was used for tumour-cell inoculation in the other animals. In Group 3 the animals were injected daily with levan administered topically from the day before the tumour inoculation. In Group 4 topical levan treatment was begun simultaneously with tumour-cell inoculation, while in Group 5 the topical levan injections were started on the day after, and in Group 6, 2 days after tumour inoculation.

Three animals of each group were killed: 4 h, 1 day, 2 days, 7 days and 14 days after inoculation of the tumour cells, and in Group 2 after the start of levan injections. The area of tumour-cell and levan inoculation was excised with a wide margin, fixed in 10% formalin and stained with haematoxylin and eosin.

### RESULTS

### *Tumour growth dependence on levan dose*

Fig. 1 shows the effect of different doses of levan on tumour size. A 10mg daily dose was more efficient than a 5mg dose. The inhibition of neoplastic growth, as judged by the average size of tumours, was 89% with 10 mg and 48% with 5 mg of levan.

### *Influence of levan on survival of tumour-inoculated mice*

Fig. 2 shows that on Day 31 after tumour-cell inoculation, when all of the untreated mice were dead, 75% of the levan-treated mice were still alive. On the day of 50% survival of the untreated mice, 90% were still alive in the treated group. 50% of the treated mice were alive and without tumours 230 days after tumour inoculation, although treatment was stopped at 116 days. The increase in life expectancy due to levan treatment was

FIG. 1.—Effect of levan dose on Lewis lung carcinoma size as a function of time. Each curve represents 10 mice. Tumour inoculation: $2 \times 10^5$ cells s.c. in back. Levan dose daily from Day 0 in region of tumour inoculation.
● = Control. ■ = Levan 5 mg. ▲ = Levan 10 mg.

FIG. 2.—Effect of levan on survival of Lewis-lung-carcinoma-inoculated mice. $2 \times 10^5$ tumour cells were inoculated into the back of each mouse on Day 0. Levan was topically injected at a daily dose of 10 mg beginning from Day 0. Each group included 8 mice.
●, Untreated. ▲, Levan-treated.

always present, but varied between experiments.

As can be seen in Fig. 2, in this experiment the incidence of tumours in the levan-treated group was 50%. In other experiments the incidence of tumours in mice treated with daily doses of 10 mg levan varied between 30 and 90%.

FIG. 3.—Effect of varying the time of beginning levan treatment on size of tumour. Each group included 10 mice. Tumour-cell inoculation as before.
□, Untreated. ▨, Treatment begun on Day 0. ▧, Treatment begun on Day 2. ⊠, Treatment begun on Day 5.

In all these experiments no metastatic spread of tumours was seen.

### *Effect of varying the time of beginning of levan treatment on tumour incidence*

Fig. 3 shows the dependence of tumour development on the time when treatment was started. Treatment begun on Day 0 was more effective than treatment started

FIG. 4.—Comparison of the effect of treatment begun before tumour inoculation on Day 0. Each group included 10 mice. Tumour and levan injection as before.
●, Untreated. ■, Treatment begun on Day − 1. ▲, Treatment begun on Day 0.

FIG. 5.—Untreated mouse of Group 1 inoculated with tumour cells 14 days earlier. Tumour mass on the left. No tissue reaction around tumour. H. & E. × 160.

FIG. 6.—Levan treatment only, 4 h after levan injection (Group 2). Amorphous mass on the left surrounded by a rim of polymorphs. H. & E. × 200.

later. On Day 10 after tumour-cell inoculation, treatment begun on Day 0 caused a 63% inhibition (as judged by tumour size) whilst treatment begun on Day 2 produced 42% inhibition, and that begun on Day 5 was almost ineffective (around Day 5 tumours were just palpable). In one experiment survival was slightly improved even when treatment was begun as late as Day 7.

FIG. 7.—Levan treatment only (Group 2). Vacuolated macrophages with early proliferation of fibroblasts and capillaries on Day 14. H. & E. ×200.

FIG. 8.—Mouse injected with levan beginning on the day before tumour-cell inoculation (Group 3) 24 h after tumour injection. On the right, polymorphs and amorphous material. On the left, large apparently undamaged tumour cells. H. & E. ×400.

Fig. 4 shows that treatment begun before tumour-cell inoculation enhanced tumour development. Mortality in animals treated from Day −1 was earlier than in untreated mice.

*Histological findings*

*Group 1: Tumour development in untreated animals.*—Four hours after tumour inoculation, the injected area contained small groups of tumour cells, the majority of which were necrotic or disintegrating. Few isolated PMNs appeared around and between tumour cells. One and 2 days after inoculation, most tumour cells were necrotic, and only few large tumour cells were seen between the necrotic debris, intermingled with a few polymorphs. At 7 and 14 days the tumours were large solid masses of pleomorphic cells with no inflammatory reaction around them (Fig. 5). The tumour infiltrated adjacent muscle and adipose tissue. Many mitotic figures were found in the tumour cells.

*Group 2: Levan treatment only.*—Four hours after s.c. injection of levan an amorphous fibrillary eosinophilic material surrounded by PMNs was seen in the lower dermis (Fig. 6). Twenty-four hours after the injection the mass of eosinophilic material was still present surrounded and infiltrated by a large number of polymorphs. In the centre of the mass there was a very dense accumulation of polymorphs. Few macrophages with abundant vacuolar cytoplasm were seen at the margin of the eosinophilic material. At Day 2 the number of polymorphs at the periphery was diminished, and many vacuolated macrophages were seen around and within the amorphous eosinophilic material. At 7 and 14 days, only a few PMNs were present and the whole area was studded with foamy macrophages. On Day 14, in addition to the macrophages, a few proliferating fibroblasts and capillaries were found (Fig. 7).

*Group 3: Topically injected levan from 1 day before tumour-cell inoculation.*—Four hours after tumour-cell inoculation a few isolated tumour cells were seen scattered between PMNs and near the amorphous material. After 1 day more isolated tumour cells were seen. Numerous PMNs infiltrated the area with no obvious signs of attraction to tumour cells. In many places the PMNs surrounded deeply stained amorphous masses which might have been dead tumour cells. Apparently undamaged tumour cells were found inside and at the periphery of the area of polymorph infiltration (Fig. 8). On Day 2 a mixture of large tumour cells, PMNs and macrophages was present. Also here the tumour cells bore no apparent relation to the infiltrate, and tumour cells adjacent to macrophages appeared undamaged (Fig. 9). On Days 7 and 14 large solid tumour masses were seen infiltrating into the neighbouring tissues, surrounded by numerous PMNs and macrophages. In the dense peripheral infiltrate of polymorphs and macrophages scattered tumour cells were visible. On Day 14 a large solid tumour mass consisting of undamaged cells was surrounded by a rim of vacuolated macrophages.

*Group 4: Topical levan treatment begun simultaneously with tumour inoculation.*—Four hours and 1 day after tumour-cell inoculation, small groups of isolated tumour cells, many of them necrotic, were seen. A heavy infiltrate of PMNs was found around and between the tumour cells, and in the eosinophilic material. Some of the tumour cells surrounded by the infiltrate appeared shrunken and homogeneously basophilic (Fig. 10). On Day 1 many vacuolated macrophages were already visible. On Day 2 the findings were similar to those of Day 1. On Day 7 only in one animal was a small group of tumour cells seen, surrounded by a necrotic area with abundant PMNs and vacuolated macrophages. In the 2 other animals no tumour cells were detected. On Day 14 small groups of tumour cells surrounded by dense infiltrates of foamy macrophages were found in 2 animals. Macrophages were also seen penetrating into the mass of tumour cells, giving them a "starry sky" appearance (Fig. 11). The tumour cells impinging on the macrophages were mostly shrunken and hyperchromatic, with no demarcation between nucleus and cytoplasm, or with relatively small hyperchromatic naked nuclei. The tumour cells not in contact with macrophages were large, with a round nucleus, 2–3 large nucleoli, and abundant basophilic cytoplasm (Fig. 12).

*Group 5: Topical levan injection starting 1 day after tumour inoculation.*—On Day 1 isolated tumour cells were seen surrounded

FIG. 9.—Mouse treated in the same way in Fig. 8, but injected 2 days after tumour-cell inoculation. A heavy infiltrate with polymorphs and macrophages. Between these cells a number of undamaged tumour cells (arrows). H. & E. × 400.

FIG. 10.—A mouse of Group 4, started on levan simultaneously with the tumour-cell inoculation, examined 1 day after inoculation. A few isolated tumour cells in the centre surrounded by massive polymorph infiltration. Some tumour cells are shrunken and basophilic. H. & E. × 200.

by polymorphs. On Day 2 and 7 dispersed tumour cells were surrounded by a heavy infiltrate of PMNs and macrophages. On Day 14 the tumour was large and solid, sharply delineated from the surrounding tissue by a wide band of vacuolated macrophages with a few polymorphs. In a few areas macrophages were seen inside the solid tumour mass. The macrophage reaction was less intense than in the mice of

FIG. 11.—Mouse of Group 4, at Day 14. Large vacuolated macrophages between tumour cells, giving the tumour a "starry sky" appearance. Tumour cells impinging on macrophages show degenerative changes which are better seen in Fig. 12. H. & E. × 200.

FIG. 12.—Same mouse as in Fig. 11. Tumour cells impinging on the large vacuolated macrophages (short arrow) are shrunken, homogeneous and hyperchromatic. Tumour cells not in contact with macrophages appear healthy (long arrow). H. & E. × 1330.

Group 4, but the changes in tumour cells impinging on macrophages were similar.

*Group 6: Topical levan injection starting 2 days after tumour inoculation.*—On Day 2 few isolated tumour cells were embedded in a mass of polymorphs. On Day 7 small groups of isolated tumour cells were surrounded by mononuclear cells and many vacuolated macrophages. On Day 14 a solid tumour mass with areas of necrosis was surrounded by a thick rim of vacuolated macrophages, many of which were seen penetrating the tumour mass. The macrophage reaction was still less than in Group 5 mice, but the changes in tumour cells in contact with macrophages were similar.

### DISCUSSION

The experimental results indicate that levan inhibited the growth of Lewis lung carcinoma, in addition to its previously reported effect on AKR lymphoma. This inhibition was manifested by a decrease in the rate of tumour growth, judged by the incidence and size of the tumours, as well as by prolonged survival of the mice.

The antitumour activity of levan could be due to one or more of the following mechanisms: (1) levan might be directly cytotoxic to the tumour cells; (2) it might induce a direct but not cytotoxic change in tumour cells, rendering them eventually more sensitive to other tumoricidal influences; (3) the polysaccharide might enhance the host's reaction against the tumour cells.

The enhancement of tumour growth in animals treated from Day −1 indicates that mechanisms other than direct cytotoxicity are operating in the antitumour effect of levan. In fact, administration of levan before tumour-cell inoculation increases the local concentration of the drug and could be expected to be more efficient than treatment begun on the day of inoculation, if a direct effect were implicated. Other studies in our laboratory have shown that levan is not directly cytotoxic to tumour cells *in vitro* (Brudner *et al.*, in preparation), although it induces a change in permeability of these cells.

S.c. injection of levan was shown to elicit an acute inflammatory reaction followed by growth of macrophage-rich granulation tissue. This is consistent with the findings of Spector *et al.* (1968), who observed a similar reaction with dextrans. Thus levan, which inhibits cellular infiltration when systemically administered, provokes such reaction by topical administration.

The present study shows that the development of Lewis lung carcinoma inoculated s.c. in mice was inhibited by topically injected levan, mainly through the host's reaction. Whereas the inoculation of tumour alone did not cause any host reaction, the levan-treated animals reacted in the first 24 h with an intense polymorphonuclear (PMN) infiltration, followed by accumulation of vacuolated macrophages. Already during the first 2 days after tumour inoculation and simultaneous levan treatment, the tumours were smaller than in the non-levanized animals, with extensive necrosis of tumour cells in the vicinity of the PMN and macrophage infiltration. This finding indicates a possibly important role of PMNs and macrophages in the defence mechanism against tumours.

Intensive PMN reaction was found by Hanna *et al.* (1972) in tumour regression after injection of BCG into the tumour. Similar findings were reported by Lieberman *et al.* (1975) after BCG injection into human malignant melanoma. Snodgrass *et al.* (1975) also described PMN infiltration into transplanted lung carcinoma treated with pyran copolymer. The precise role of PMNs in tumour-cell destruction is unknown. Clark & Klebanoff (1975) demonstrated a cytotoxic effect of human PMNs on neoplastic cells. These authors postulated killing of tumour cells by the peroxidase system. Clark *et al.* (1975) and Strauss *et al.* (1974) showed an inhibitory effect of blood-cell peroxidase systems on tumour cells.

Numerous authors claim that histiocytes are the major effector cells in tumour regression. The mechanism by which macrophages destroy tumour cells is not quite clear. Hanna *et al.* (1972) and Lieberman *et al.* (1975) suggest that BCG activates both specific and nonspecific immune responses, which increase age killing of tumour cells by activated macrophages. Feldman *et al.* (1974) showed phagocytosis of tumour cells by macrophages. Chambers & Weiser (1969) also showed that activated macrophages phagocytose portions of tumour cells.

Another mechanism for the cytotoxicity of activated macrophages against tumour cells was suggested by Hibbs (1974) who demonstrated transfer of lysosomes to the tumour cells from activated macrophages. Snodgrass & Hanna (1973) showed that tumour cells were killed when they were in close contact with the plasma membrane of BCG-activated macrophages. A similar finding was reported by Snodgrass *et al.* (1975) who demonstrated that intimate contact between pyran-activated histiocytes and carcinoma cells was associated with degeneration of the tumour cells.

Cells of Lewis lung carcinoma were shown by Fauve *et al.* (1974) to be able to repulse macrophages *in vitro*. Levan seems to overcome this property of the Lewis lung carcinoma cells, since macrophages were seen to approach tumour cells. Histological examination showed vacuolated levan-laden macrophages encircling and penetrating the tumour masses. Tumour cells in close contact with the activated macrophages showed shrunken nuclei and cell bodies, and various stages of necrosis. Tumour cells not in close contact with macrophages appeared unchanged. No phagocytosis of tumour cells by macrophages was seen. Lately, Bomford & Moreno (1977) showed an *in vitro* antitumour effect of cytostatic macrophages induced by levan. They actually showed that macrophages activated by low doses of levan had an antitumour effect, but practically only *in vitro*. A stimulated macrophage reaction around the tumour was found for other polysaccharides by Tokuzen (1973). Another, non-polysaccharide polymer, pyran, has been shown by Schultz *et al.* (1977) to have a similar effect.

Our findings are in agreement with the studies of Snodgrass & Hanna (1973) and Snodgrass *et al.* (1975) showing intimate contact between activated macrophages and tumour cells. This conclusion is supported by yet unpublished results which show that macrophages harvested from the peritoneum of levan-treated mice delay tumour appearance. Levan seems to represent an efficient activator of macrophages.

The administration of levan beginning on the day before tumour-cell inoculation was shown to enhance tumour growth. A similar effect of preliminary treatment with levan was found in the AKR lymphoma (Sinai *et al.*, 1976). The microscopic findings described here might contribute to the understanding of this phenomena; although levan injection produced a PMN and macrophage response, the cells appeared not to have been attracted to the tumour cells and not to damage them.

In addition, levan administered before the injection of tumour cells may block the RES. Impairment of macrophage function was shown by others to enhance tumour growth (Isa & Sanders, 1975).

This study was in part supported by a grant from the Israel Cancer Society.

### REFERENCES

ALGIRE, G. H., LEGALLAIS, F. Y. & ANDERSON, B. F. (1952) Vascular reactions of normal and malignant tissues *in vivo*. V. The role of hypotension in the action of a bacterial polysaccharide on tumours. *J. Natl Cancer Inst.*, **12**, 1279.

BEHAR, A. & SHILO, M. (1969) Effect of native levan on membrane permeability: I. Effect on the vascular membrane in acute non-specific inflammation. A study with the light microscope. *Am. J. Pathol.*, **57**, 383.

BELKIN, M., HARDY, W. G., PERRAULT, A. & SATO, H. (1959) Swelling and vacuolization induced in ascites tumour cells by polysaccharides of higher plants. *Cancer Res.*, **19**, 1050.

BERMAN, Z., LEIBOVICI, J. & WOLMAN, M. (1976) Effect of levan on the stages of development of experimental allergic encephalomyelitis. *Israel J. Med. Sci.*, **12**, 1294.

BOMFORD, R. & MORENO, C. (1977) Mechanism of the anti-tumour effect of glucans and fructosans: a comparison with *C. parvum*. *Br. J. Cancer*, **36**, 41.

CHAMBERS, V. C. & WEISER, R. S. (1969) The ultrastructure of target cells and immune macrophages during their interaction *in vitro*. *Cancer Res.*, **29**, 301.

CLARK, R. A. & KLEBANOFF, S. J. (1975) Neutrophil-mediated tumour cell cytotoxicity: role of the peroxidase system. *J. Exp. Med.*, **141**, 1442.

CLARK, R. A., KLEBANOFF, S. J., EINSTEIN, A. B. & FEFFER, A. (1975) Peroxidase-$H_2O_2$-halide system: cytotoxic effect on mammalian tumour cells. *Blood*, **45**, 161.

COUTINHO, A. & MOLLER, G. (1973) B cell mitogenic properties of thymus independent antigens. *Nature* (*New Biol.*), **245**, 12.

DAVIES, A. M., SHILO, M. & HESTRIN, S. (1955) The influence of Aerobacter levan on the permeability of the blood vessels of the skin: studies with antibody globulins and trypan blue. *Br. J. Exp. Pathol.*, **36**, 500.

FAUVE, R. M., HEVIN, B., JACOB, H., GAILLARD, J. A. & JACOB, F. (1974) Anti-inflammatory effects of murine malignant cells. *Proc. Natl Acad. Sci. U.S.A.*, **71**, 4052.

FELDMAN, D. G., EHRENREICH, T. & GROSS, L. (1974) Electron microscopic study of the growth and regression of leukemic intradermal tumours in guinea pigs. *Cancer Res.*, **34**, 901.

HANNA, M. D., ZBAR, B. & RAPP, H. J. (1972) Histopathology of tumour regression after intralesional injection of *Mycobacterium bovis*. I. Tumour growth and metastasis. *J. Natl Cancer Inst.*, **48**, 1441.

HESTRIN, S., SHILO, M. & FEINGOLD, D. S. (1954) Infection-promoting activity of levan and dextran as a function of degree of polymerization. *Br. J. Exp. Pathol.*, **35**, 107.

HIBBS, J. B., JR (1974) Discrimination between neoplastic and non-neoplastic cells *in vitro* by activated macrophages. *J. Natl Cancer Inst.*, **53**, 1487.

HOENIG, S., KAZAP, I. & LEIBOVICI, J. (1978) Suppression of humoral immune response in mice by administration of high molecular levan. *Experientia*, **34**, 1362.

ISA, A. M. & SANDERS, B. R. (1975) Enhancement of tumour growth in allogeneic mice following impairment of macrophage function. *Transplantation*, **20**, 296.

LAZAR, A. & LAZAR, D. C. (1962) Effect of methylcellulose on the Murphy-Sturm lymphosarcoma in rats. *J. Natl Cancer Inst.*, **28**, 1255.

LEIBOVICI, J., BLEIBERG, I. & WOLMAN, M. (1975) Effect of native levan on homograft rejection in mice. *Proc. Soc. Exp. Biol. Med.*, **149**, 348.

LEIBOVICI, J., SINAI, Y., WOLMAN, M. & DAVIDAI, G. (1975) Effect of high-molecular levan on the growth and spread of AKR lymphoma. *Cancer Res.*, **35**, 1921.

LIEBERMAN, R., WYBRAN, J. & EPSTEIN, E. (1975) The immunologic and histopathologic changes of BCG-mediated tumour regression in patients with malignant melanoma. *Cancer*, **35**, 766.

MAEDA, Y. Y. & CHIHARA, G. (1971) Lentinan, a new immuno-accelerator of cell-mediated responses. *Nature*, **229**, 634.

ROBERTSON, T. A., PAPADIMITRIOU, J. M., WALTERS, M. N-I. & WOLMAN, M. (1977) Effects of exposure of murine peritoneal exudate and resident macrophages to high molecular levan: a morphological study. *J. Pathol.*, **123**, 157.

ROE, E. M. F., SMITH, H. & FLOHAVAN, E. (1972) Action of tumour-inhibitory gum tragacanth on potassium permeability of ascites tumour cells and partial characterization of the cytotoxic component. *Cancer Res.*, **32**, 2067.

SCHULTZ, R. M., PAPAMATHEAKIS, J. D., LUETZELLER, J., RUIZ, P. & CHIRIGOS, M. A. (1977) Macrophage involvement in the protective effect of pyran copolymer against the Madison lung carcinoma (M109). *Cancer Res.*, **37**, 358.

SHEZEN, E., LEIBOVICI, J. & WOLMAN, M. (1978). Modification of the tuberculin reaction by levan. *Br. J. Exp. Pathol.*, **59**, 454.

SHILO, M., WOLMAN, M. & WOLMAN, B. (1956) Inhibition of inflammatory response of the skin to *Staphylococcus aureus* by high molecular levan. *Br. J. Exp. Pathol.*, **37**, 219.

SINAI, Y., LEIBOVICI, J. & WOLMAN, M. (1976) Effect of route and schedule of administration of high-molecular levan on the growth of AKR lymphoma. *Cancer Res.*, **36**, 1593.

SNODGRASS, M. J. & HANNA, M. G. (1973) Ultrastructural studies of histiocyte-tumour cell interaction during tumour regression after intralesional injection of *Mycobacterium bovis*. *Cancer Res.*, **33**, 701.

SNODGRASS, M. J., MORAHAN, P. S. & KAPLAN, A. M. (1975) Histopathology of the host response to Lewis lung carcinoma: modulation by pyran. *J. Natl Cancer Inst.*, **55**, 455.

SPECTOR, W. G., HEESOM, N. & STEVENS, J. E. (1968) Factors influencing chronicity in inflammation of rat skin. *J. Pathol.*, **96**, 203.

STRAUSS, R. R., PAUL, B. B., SELVARAJ, R. J. & SBARRA, A. J. (1974) A peroxidase inhibitor in leukaemic AKR mouse spleen cells. *Cancer Res.*, **34**, 3220.

TOKUZEN, R. (1973) Comparison of local cellular reaction to tumour grafts in mice treated with some plant polysaccharides. *Cancer Res.*, **31**, 1590.

WHISTLER, R. L., BUSHWAY, A. A., SINGH, P. B., NAKAHARA, W. & TOKUZEN, R. (1976) Noncytotoxic, antitumour polysaccharides. *Adv. Carbohydrate Chem. Biochem.*, **32**, 235.

WOLMAN, M. (1956) Histological changes produced by injections of polysaccharides. *Arch. Pathol.*, **62**, 149.

WOLMAN, M. & WOLMAN, B. (1956) Effect of polysaccharides on the formation of granulation tissue. *Arch. Pathol.*, **62**, 74.